# Alternative Attractors of Shallow Lakes

**DOI:** 10.1100/tsw.2001.62

**Published:** 2001-07-17

**Authors:** Marten Scheffer

**Affiliations:** Department of Aquatic Ecology and Water Quality Management, Wageningen University, P.O. Box. 8080, NL-6700 DD Wageningen, The Netherlands

**Keywords:** lake, eutrophication, fish, aquatic vegetation, macrophytes, plankton, Daphnia, phytoplankton, alternative stable states, regime shifts, catastrophe theory, model, predation, trophic cascades, top-down control, turbidity, stability, resilience, hysteresis, bifurcation, multiple attractors

## Abstract

Ponds and shallow lakes can be very clear with abundant submerged plants, or very turbid due to a high concentration of phytoplankton and suspended sediment particles. These strongly contrasting ecosystem states have been found to represent alternative attractors with distinct stabilizing feedback mechanisms. In the turbid state, the development of submerged vegetation is prevented by low underwater light levels. The unprotected sediment frequently is resuspended by wave action and by fish searching for food causing a further decrease of transparency. Since there are no plants that could serve as refuges, zooplankton is grazed down by fish to densities insufficient to control algal blooms. In contrast, the clear state in eutrophic shallow lakes is dominated by aquatic macrophytes. The submerged macrophytes prevent sediment resuspension, take up nutrients from the water, and provide a refuge for zooplankton against fish predation. These processes buffer the impacts of increased nutrient loads until they become too high. Consequently, the response of shallow lakes to eutrophication tends to be catastrophic rather than smooth, and various lakes switch back and forth abruptly between a clear and a turbid state repeatedly without obvious external forcing. Importantly, a switch from a turbid to a stable clear state often can be invoked by means of biomanipulation in the form of a temporary reduction of the fish stock.

## INTRODUCTION

For most of its history, limnology has focused on deep lakes that stratify in summer. This stratification largely isolates the upper water layers (epilimnion) from the colder, deep water (hypolimnion) and from interaction with the sediment during the summer. The impact of macrophytes on the community is relatively small in such lakes, as plant growth is restricted to a relatively narrow marginal zone. In contrast, shallow lakes can be largely colonized by macrophytes and do not stratify for long periods in summer. This type of lake, where the entire water column is frequently mixed, is referred to as polymictic. The average depth of many of these lakes is less than 3 m, but their surface area ranges from <1 ha to >100 km$^2$. Obviously, the intense sediment-water interaction and the potentially large impact of aquatic vegetation makes the functioning of shallow lakes different from that of their deep counterparts in many aspects.

In several parts of the world shallow lakes are more abundant than deep ones. Numerous shallow lakes, for instance, are found at the edge of the ice cover during the Weichselian glaciation period. Also, human activities such as digging for peat, sand, gravel, or clay have produced considerable numbers of shallow lakes and ponds. The term wetlands is often used to refer to shallow lakes and adjacent marshy land. Such habitats are notoriously rich in wildlife. In densely populated areas even small lakes can be very important from a recreational point of view. Fishing, swimming, boating and bird watching attract a large public.

The pristine state of the most shallow lakes is probably one of clear water and a rich aquatic vegetation. Nutrient loading has changed this situation in many cases. The lakes have shifted from clear to turbid, and with the increase in turbidity, submerged plants have largely disappeared. Efforts to restore the clear state by means of reduction of the nutrient loading often are unsuccessful. This has invoked experiments with additional methods such as temporary reduction of the fish stock. This has catalyzed the development of insights into the mechanisms that govern the dynamics of shallow lake communities. A full account of the brief overview presented here can be found in Reference [1] (http://www.slm.wau.nl/wkao/). The key references to the theory of alternative equilibria in shallow lakes are found in References [2] and [3].

## HOW NUTRIENT LOADING MAKES LAKES TURBID

The sequence of changes in shallow lakes during eutrophication is not well documented, but some elements are agreed upon by most workers in the field[4]. Shallow lakes with a low nutrient content usually have a vegetation dominated by relatively small plants. With increased nutrient loading the biomass of aquatic macrophytes increases and plants that fill the entire water column or concentrate much of their biomass in the upper water layer become dominant. Such dense weedbeds are often experienced as a nuisance by the fishing and boating public. When weed control programs eradicate the vegetation, turbidity in shallow lakes tends to increase strongly due to algal blooms and wind resuspension of the sediment. Also, when vegetation is not controlled explicitly, further eutrophication of vegetated lakes can lead to a gradual increase of phytoplankton biomass and of the periphyton layer that covers the plants. Shading by these organisms ultimately leads to a collapse of the vegetation due to light limitation.

Restoration of non-vegetated turbid shallow lakes to the clear vegetated state is notoriously difficult. Reduction of external nutrient loading may have little effect, as during the period of eutrophication a large amount of phosphorus has often been adsorbed by the sediment. When the loading is reduced and its concentration in the water drops, phosphorus release from the sediment becomes an important nutrient source for phytoplankton. Thus a reduction of the external loading is often compensated by ‘internal loading’, delaying the response of the lake water concentration to the reduction of external loading[5,6].

## FEEDBACKS MAINTAINING THE TURBID STATE

Internal loading is not the only reason why restoration of turbid shallow lakes is difficult. With the disappearance of aquatic vegetation the structure of the shallow lake community changes dramatically (Fig. 1). Invertebrates that are associated with vegetation disappear and with these animals the birds and fishes that feed on them or on the plants[7]. Also, vegetation provides an important refuge against predation for many animals, and hence its disappearance causes crucial shifts in many predator-prey relationships. Large zooplankton use vegetation as a daytime refuge against fish predation[8,9]. In vegetated lakes they can contribute significantly to the control of phytoplankton biomass. In the absence of vegetation their numbers are strongly reduced. This, and the increased nutrient availability allows phytoplankton biomass to be higher in the absence of vegetation. In addition, wave resuspension of the unprotected sediment can cause a considerable additional turbidity once the vegetation has vanished[10,11]. The fish community of unvegetated lakes becomes dominated by species that forage on benthic invertebrates[12]. The activity of fish promotes the nutrient flux from the sediment into the water and causes an extra resuspension of sediment particles, contributing to the already high turbidity[13].

Return of submerged plants in this situation is unlikely, in part because their absence has allowed a further increase in turbidity, but also because the frequent disturbance of sediment by wind and benthivorous fish hampers resettlement[1]. Ecological feedback mechanisms are thus an important reason why restoration of the vegetated clear water state is difficult. Strong measures such as removal of part of the fish stock and changes in the water level have been used successfully as a way to break the feedback that keeps such lakes turbid.

## FEEDBACKS MAINTAINING A CLEAR STATE

It has long been noted that water tends to be less turbid if there is aquatic vegetation. The first publications result from work in fish culture ponds[14] where the phytoplankton density was lowest in years with high aquatic macrophyte abundance. Many later studies report an enhanced water clarity in the presence of vegetation. Analyses of the relationship between transparency and the macrophyte abundance in large sets of lakes confirm that there is a systematic correlation. Danish work[15], for example, indicates that lakes with dense submerged macrophytes have a higher transparency than comparable lakes that lack such vegetation. Proof that a negative effect of vegetation on phytoplankton biomass is partly responsible for the correlation between clarity and vegetation was obtained experimentally over 50 years ago[16].

The picture emerging from many case studies is that shading[17], reduction of nutrient availability[18,19], excretion of allelopathic substances[20,21], reduction of resuspension[11,22], and enhanced grazing[9,23] may contribute to the effect of aquatic macrophytes on water clarity, although the relative importance of each of these factors varies considerably from case to case.

Since light is a main limiting factor for submerged macrophyte growth in turbid lakes the clearing effect of plants on water implies a positive feed-back in the development of submerged vegetation: once they grow, the water clears up and they grow even better. Fig. 2 summarizes the main mechanisms involved. A simple way of evaluating the overall effect of the depicted interactions is to multiply the signs along the way of a path through the scheme. This exercise shows that through all depicted routes turbidity enhances turbidity, and vegetation enhances vegetation.

## A GRAPHICAL MODEL

The existence of stabilizing mechanisms that tend to keep the system in either a vegetation- or phytoplankton-dominated state suggests the potential for alternative stable states.

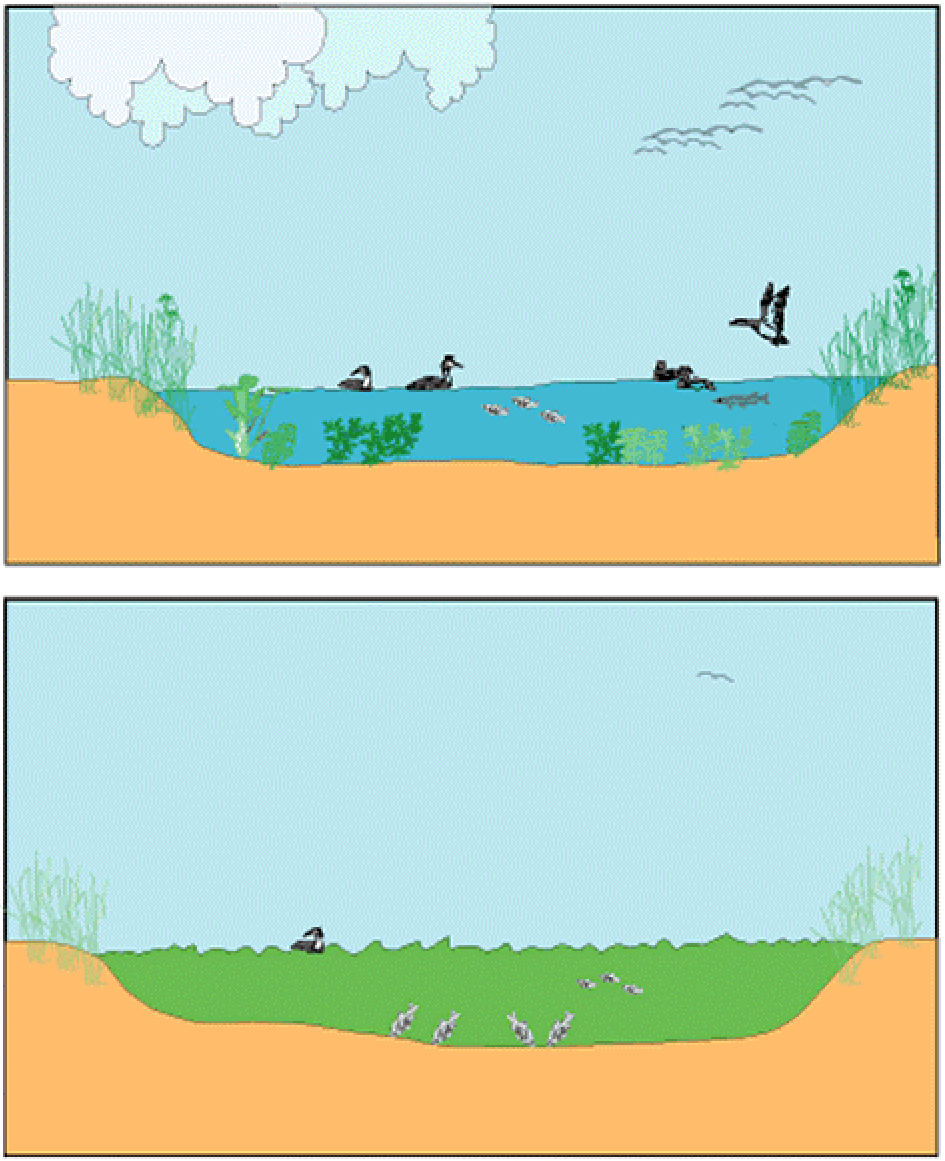

**FIGURE 1.** Schematic representation of a shallow lake in a vegetation-dominated clear state (upper panel) and in a turbid phytoplankton-dominated state in which submerged vegetation is largely absent and fish and waves stir up the sediments.

However, in mathematical models alternative equilibria usually occur for limited ranges of parameter settings only, and likewise, real systems will normally have these properties only for a limited set of conditions. Indeed, the hypothesized stabilization of the vegetated state seems unlikely in deep lakes where the narrow littoral zone that can be vegetated has a less dramatic impact on turbidity than in shallow lakes that can be entirely vegetated. Also in shallow lakes the existence of alternative stable states will be limited to an intermediate range of nutrient levels, as oligotrophic lakes are rarely turbid and very high nutrient loading usually excludes vegetation dominance. Therefore, the demonstration of stabilizing mechanisms per se is not sufficient to conclude that a lake has alternative stable states.

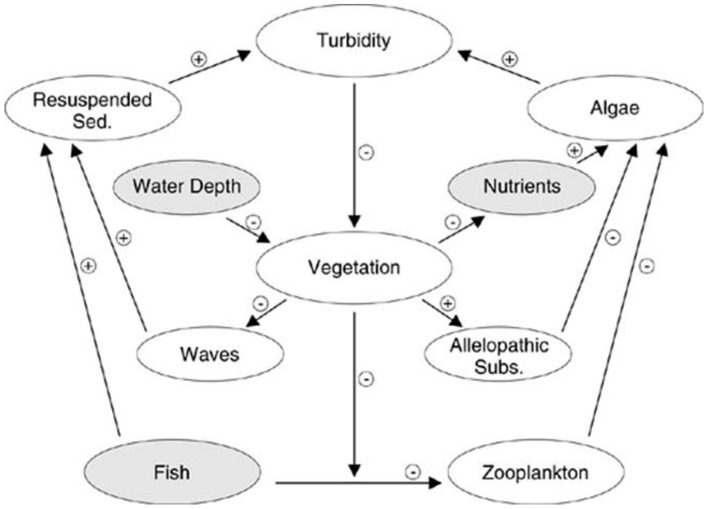

**FIGURE 2.** Feedbacks that may cause a vegetation dominated state and a turbid state to be alternative equilibria. The qualitative effect of each route in the diagram can be computed by multiplying the signs along the way. This shows that both the vegetated and the turbid state are self-reinforcing.

A simple graphical model suffices to clarify roughly how nutrient loading and water level may affect the stability properties of the ecosystem. The model is based on three assumptions:

1. Turbidity increases with increasing nutrient levels.
2. Vegetation reduces turbidity.
3. Vegetation disappears entirely when a critical turbidity is exceeded.

In view of the first two assumptions, equilibrium turbidity can be drawn as two different functions of the nutrient level (Fig. 3): one for a macrophyte dominated, and one for an unvegetated situation. Above a critical turbidity, macrophytes will be absent, in which case the upper equilibrium line is the relevant one; below this turbidity the lower equilibrium curve applies. Over a range of intermediate nutrient levels two alternative equilibria exist: one with macrophytes, and a more turbid one without vegetation. At lower nutrient levels, only the macrophyte-dominated equilibrium exists, whereas at the highest nutrient levels, there is only a vegetationless equilibrium.

The course of the eutrophication process can be derived from this picture. Gradual enrichment starting from low nutrient levels will cause the system to proceed along the lower equilibrium curve until the critical turbidity is reached at which macrophytes disappear. A jump to a more turbid equilibrium at the upper part of the curve occurs. In order to restore the macrophyte-dominated state by means of nutrient management, the nutrient level must be lowered to a value where algal growth is limited enough by nutrients alone to reach the critical turbidity for macrophytes again. At the extremes of the range of nutrient levels over which alternative stable states exist, either of the equilibrium lines approaches the critical turbidity that represents the breakpoint of the system. This corresponds to a decrease of stability. Near the edges, a small perturbation is enough to bring the system over the critical line and to cause a switch to the other equilibrium.

Water level in the lake is another important control variable with respect to aquatic macrophyte dominance. Since vegetation can resist a higher turbidity if the lake is shallower, the horizontal breakpoint-line in the diagram will be at a higher critical turbidity in shallower lakes. It can be seen from the graphical model that a small shift in critical turbidity resulting from a change in water level can bring about a switch from one state to the other in lakes that are close to the breakpoint already. This is in line with observations in several lakes[24,25,26].

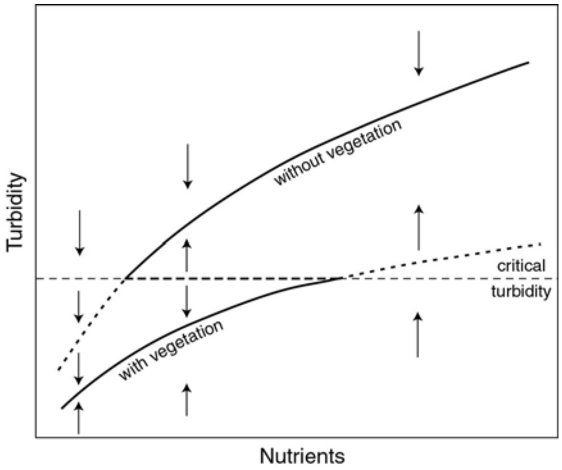

**FIGURE 3.** Alternative equilibrium turbidities caused by disappearance of submerged vegetation when a critical turbidity is exceeded (see text for explanation). The arrows indicate the direction of change when the system is not in one of the two alternative stable states.

## A MATHEMATICAL MODEL

It is useful for various analyses to also capture the basic properties of the catastrophic response of ecosystems in a mathematical model. Although an impressive number of empirical studies supports the idea that hysteresis is a common property of shallow lake ecosystems, and there is abundant evidence for the existence of mechanisms that tend to cause a positive feedback in the development of aquatic vegetation, our quantitative insight into the functioning of the system is still surprisingly poor. Indeed, we are still far from being able to produce a detailed mechanistic model that incorporates the discussed mechanisms in a way that allows us to predict whether a given lake will possess alternative clear and turbid equilibria and for which set of conditions.

Nonetheless, on a higher abstraction level the dynamic properties of this and many other ecosystems may well be captured by a single differential equation. As an example consider an equation describing the change over time of an ‘unwanted’ ecosystem property *x*:

$$dx/dt=a - b\,x + r\,f(x) \qquad (1)$$

The parameter *a* represents stress imposed by human use which promotes *x*. The remainder of the equation describes the internal dynamics: parameter *b* represents the rate at which *x* decays in the system whereas *r* is the rate at which *x* recovers again as a function *f* of *x*. For lakes one can think of *x* as nutrients suspended in phytoplankton causing turbidity, of *a* as nutrient loading, of *b* as nutrient removal rate, and of *r* as internal nutrient recycling. This specific equation has also been proposed to mimic the dynamics of nutrient loaded deep lakes[27].

For $r = 0$ , the model has a single equilibrium at $x = a/b$. The last term, however, can cause the existence of alternative stable states, for instance, if $f(x)$ is a function that increases steeply at a threshold ($h$), as in the case of the Hill function: $f(x) = x^p/(x^p+h^p)$ where the exponent $p$ determines the steepness of the switch occurring around $h$. Notice that (1) can only have multiple stable states if the maximum $\{rf'(x)\} > b$. Thus, steeper Hill functions (resulting from higher $p$ values) have higher chances of resulting in alternative stable states.

An advantage of the high abstraction level of minimal models such as the proposed one is that they are very general. For instance, one may imagine model (1) to describe desertification interpreting *x* as barren soil, *a* as vegetation destruction, *b* as recolonization of barren soil by plants, and *r* as erosion by wind and run-off[28].

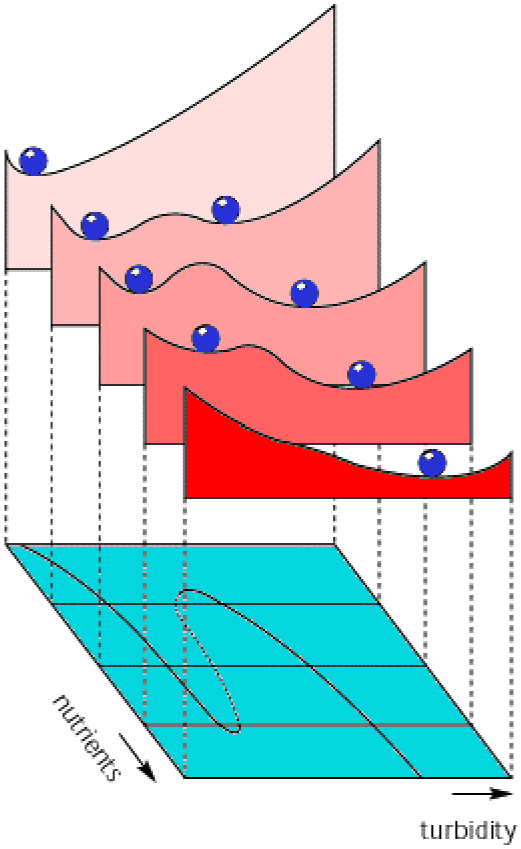

**FIGURE 4.** 'Marble-in-a-cup' representation of the stability properties of lakes at five different levels of nutrient loading. (See text).

Although the simplicity of the above model may give the impression that it cannot capture well what goes on in shallow lakes, more elaborate and complex simulation models tend to produce the same pattern of alternative stable states[29].

## STABILITY PROPERTIES

The overall stability properties of models of shallow lakes with alternative stable states can be summarized in a simple and intuitively straightforward way by means of a 'stability landscape' or 'marble-in-a-cup diagram' (Fig. 4). The system, like a ball, tends to move downhill and settle in the deepest point that is an equilibrium. The slope of the surface determines the direction and speed of movement. Such a stability landscape can be computed from a mathematical model of the system by using the derivative as the slope of the hills in the stability landscapes. As indicated in the figure they correspond to a a sigmoidal hysteresis curve which is a smooth-edged version of the graphically derived model presented earlier (Fig. 3). On the hilltops and in the deepest point of the valleys, the slope is zero, corresponding to a derivative of zero and thus to an equilibrium. However, only the minimum of the stability landscapes represents stable equilibria. The hilltops are unstable equilibria and represent the breakpoints that mark the limits of the basins of attraction of the stable equilibria.

With respect to the response of the lake to management it is important to distinguish between disturbances and measures that affect the stability properties. In terms of stability landscapes (Fig. 4) disturbances are displacements of the ball but do not alter the pattern of hills and valleys. Fish-kills, herbicide treatments, and heavy storms are examples. If there is only one stable state (valley), the effect of a disturbance will be temporary as the system will settle to this same state again. However, if two alternative stable states (valleys) are present, the system may settle to the alternative stable state if the disturbance has been strong enough to move it past the breakpoint (hilltop). On the contrary, gradual changes in external conditioning factors such as the nutrient loading or the average water level of the lake will change the stability properties (the landscape in Fig. 4) which may also cause a shift but has distinctly different implications with respect to management than disturbances.

Changes in the nutrient loading are probably a major reason why the stability properties of shallow lakes have changed (Fig. 4). At low nutrient levels, the system has one globally stable equilibrium, a clear water state. Increase of the nutrient level gradually changes the shape of the stability landscape, and gives rise to an alternative turbid equilibrium. However, if no major disturbances occur, the system will stay in its current state, responding only weakly to the enrichment. If the nutrient level is raised further, the stability of the clear state decreases, and slight perturbations are enough to cause a switch to the turbid equilibrium. At still higher nutrient levels the clear equilibrium disappears. This inevitably leads to an irreversible jump to the turbid state. Efforts to restore the system by reducing the nutrient level will change the stability landscape again, but even if nutrient levels at which the system was formerly clear are realized, the response may be minor (Fig. 5). Despite the reappearance of an alternative clear equilibrium, the locally stable turbid state tends to be sustained. Only a drastic reduction of nutrient level will be sufficient to make the turbid state unstable, resulting in a switch to the clear state.

The two alternative equilibria are not equally likely to be found when they both exist. The probability of ending up in either of the equilibria in an environment with frequent stochastic events depends on the stability properties of the system, e.g., the size of the basins of attraction of the two alternative equilibria. The breakpoint (hilltop) moves toward either of the stable equilibria if the nutrient level changes, indicating that the robustness of vegetated equilibrium decreases with the nutrient level, while that of the turbid state increases with enrichment. As a result the vegetated state should be expected to be more robust to disturbances at lower nutrient values, whereas the system is more likely to settle to the turbid state when nutrient loading is higher. Similarly, disturbance-type measures are more likely to lead to a vegetated clear-water state if the nutrient loading is lower.

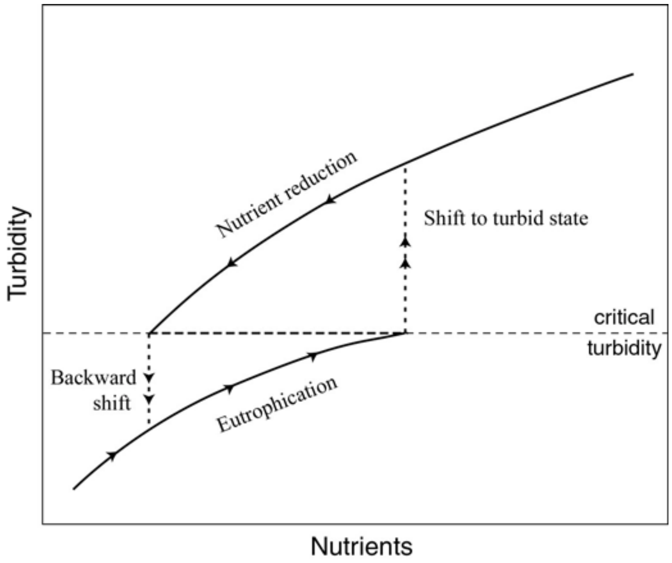

**FIGURE 5.** As a consequence of the alternative stable states the lake shows hysteresis in response to changes in nutrient loading. Once the lake has shifted to the turbid state, a reduction of nutrients leads to a relatively moderate decrease in turbidity along the upper branch of the equilibrium graph until nutrient levels have dropped enough to go through a backward switch.

Importantly, analyses with a variety of models indicate that alternative stable states will not always be present. The degree of hysteresis is predicted to be especially sensitive to the depth profile of a lake. In deep lakes no hysteresis at all is thought to occur. It has been hypothesized, however, that deep lakes may have hysteresis in their response to nutrient loading due to a positive feedback in phosphorus release from their sediments with high algal biomass[27]. Also in shallow lakes other mechanisms may lead to shifts between alternative stable states. For instance, dominance by filamentous cyanobacteria of the *Oscillatoria* type is likely to represent an alternative stable state to other phytoplankton communities in shallow lakes, due to the fact that these cyanobacteria promote turbidity but are also the better competitors under turbid conditions[30]. Although interesting patterns have been revealed, we are clearly only beginning to understand the implications of feedbacks in aquatic ecosystems that may lead to multiple attractors.
